# Safety and feasibility of focused ultrasound neuromodulation in temporal lobe epilepsy

**DOI:** 10.1186/2050-5736-3-S1-O27

**Published:** 2015-06-30

**Authors:** Alexander Bystritsky, Alexander Korb, John Stern, Mark Cohen

**Affiliations:** 1 University of California at Los Angeles, Los Angeles, CA, United States

## Background/introduction

Temporal lobe epilepsy (TLE) is the most common pharmacologically refractory form of epilepsy. While it can often be effectively treated by temporal lobe surgery, that does not always eliminate seizures, and many patients are not suitable candidates for surgery. A non-invasive method to augment surgical or medical treatment of TLE would be highly useful. Low-intensity focused ultrasound pulsation (LIFUP) offers the potential for non-invasive neuromodulation, and in animal studies has not shown any evidence of tissue damage. However, the technology has not yet been tested in humans. We are currently testing the safety and feasibility of using a LIFUP device on humans to modulate brain activity in the temporal lobe.

## Methods

Participants will be recruited from patients with temporal lobe epilepsy treated by the UCLA Seizure Disorder Center’s who have elected to undergo temporal lobe surgery. In the week prior to the scheduled surgery participants will undergo simultaneous LIFUP and fMRI using various LIFUP pulsing paradigms to excite or suppress neural tissue. The BOLD signal will be analyzed to determine whether LIFUP can activate or suppress region-specific neural activity in the temporal lobe. LIFUP will be administered at 3 different intensities.

To help determine safety of LIFUP participants will also undergo pre- and post-EEG and neuropsychological testing. To determine whether or not LIFUP causes tissue damage, post- surgery samples of the resected temporal lobe tissue in both sonicated and un-sonicated areas will undergo histological analysis.

